# Clinical evaluation of an artificial intelligence-assisted cytological system among screening strategies for a cervical cancer high-risk population

**DOI:** 10.1186/s12885-024-12532-y

**Published:** 2024-06-27

**Authors:** Wen Yang, Xiangshu Jin, Liying Huang, Shufang Jiang, Jia Xu, Yurong Fu, Yaoyao Song, Xueyan Wang, Xueqing Wang, Zhiming Yang, Yuanguang Meng

**Affiliations:** 1 Department of Obstetrics and Gynecology, the Seventh Medical Center of Chinese PLA General Hospital, Beijing, China; 2 Tianjin Central Hospital of Gynecology Obstetrics, Tianjin, China; 3 Department of Obstetrics and Gynecology, the First Medical Center of Chinese PLA General Hospital, Beijing, China; 4 iDeepWise Artificial Intelligence Robot Technology (Beijing) Co., LTD, 12 Shangdi Xinxin Road, Beijing, China; 5 School of Medicine, Nankai University, Tianjin, China

**Keywords:** Artificial intelligence-assisted cytology (AI), Cervical cancer screening, Low-grade squamous intraepithelial lesion (LSIL), High-grade squamous intraepithelial lesion (HSIL), Liquid-based cytology (LBC), Human papillomavirus (HPV)

## Abstract

**Background:**

Primary cervical cancer screening and treating precancerous lesions are effective ways to prevent cervical cancer. However, the coverage rates of human papillomavirus (HPV) vaccines and routine screening are low in most developing countries and even some developed countries. This study aimed to explore the benefit of an artificial intelligence-assisted cytology (AI) system in a screening program for a cervical cancer high-risk population in China.

**Methods:**

A total of 1231 liquid-based cytology (LBC) slides from women who underwent colposcopy at the Chinese PLA General Hospital from 2018 to 2020 were collected. All women had received a histological diagnosis based on the results of colposcopy and biopsy. The sensitivity (Se), specificity (Sp), positive predictive value (PPV), negative predictive value (NPV), false-positive rate (FPR), false-negative rate (FNR), overall accuracy (OA), positive likelihood ratio (PLR), negative likelihood ratio (NLR) and Youden index (YI) of the AI, LBC, HPV, LBC + HPV, AI + LBC, AI + HPV and HPV Seq LBC screening strategies at low-grade squamous intraepithelial lesion (LSIL) and high-grade squamous intraepithelial lesion (HSIL) thresholds were calculated to assess their effectiveness. Receiver operating characteristic (ROC) curve analysis was conducted to assess the diagnostic values of the different screening strategies.

**Results:**

The Se and Sp of the primary AI-alone strategy at the LSIL and HSIL thresholds were superior to those of the LBC + HPV cotesting strategy. Among the screening strategies, the YIs of the AI strategy at the LSIL + threshold and HSIL + threshold were the highest. At the HSIL + threshold, the AI strategy achieved the best result, with an AUC value of 0.621 (95% CI, 0.587–0.654), whereas HPV testing achieved the worst result, with an AUC value of 0.521 (95% CI, 0.484–0.559). Similarly, at the LSIL + threshold, the LBC-based strategy achieved the best result, with an AUC of 0.637 (95% CI, 0.606–0.668), whereas HPV testing achieved the worst result, with an AUC of 0.524 (95% CI, 0.491–0.557). Moreover, the AUCs of the AI and LBC strategies at this threshold were similar (0.631 and 0.637, respectively).

**Conclusions:**

These results confirmed that AI-only screening was the most authoritative method for diagnosing HSILs and LSILs, improving the accuracy of colposcopy diagnosis, and was more beneficial for patients than traditional LBC + HPV cotesting.

## Background

Cervical cancer (CC) is a preventable disease if human papillomavirus (HPV) vaccines are widely used, cervical dysplasia lesions are detected early, and patients are treated adequately. HPV persistence is the main factor influencing the risk of developing HPV-related diseases, including cervical dysplasia and CC [1]. The World Health Organization has adopted global strategies to accelerate the elimination of CC as a public health problem by 2030. This includes a goal of vaccinating 90% of girls before 15 years of age, screening 70% of women with at least 2 high-precision tests before 45 years old, and identifying 90% of women that could be diagnosed with cervical precancerous lesions or cancer for treatment [2]. To date, CC remains the most common gynaecological cancer and is the leading cause of morbidity and mortality among young women worldwide. In 2020, there were an estimated 604 127 new CC cases annually and 341 831 CC-related deaths per year worldwide [3]. This is due to poor access to screening and treatment services, especially for women living in low- and middle-income countries. Although advanced CC patients have a poor prognosis, early-stage CC patients can achieve good survival outcomes with proper treatment. This disease can manifest in a severe form but is susceptible to highly effective treatment, especially when it is detected early through prevention strategies, early diagnosis and appropriate therapies [4]. Thus, CC screening remains an effective way to prevent the disease in high-risk populations, such as those with high-risk HPV infections, low-grade squamous intraepithelial lesions (LSILs), high-grade squamous intraepithelial lesions (HSILs), atypical squamous cells – cannot exclude high grade squamous intraepithelial lesions (ASC-H), atypical glandular cells (AGC), invasive squamous cell carcinoma, adenocarcinoma in situ (AIS) and adenocarcinoma referred for colposcopy with histology as stratified by biopsy results.

Currently, three primary screening strategies are recommended for triaging patients to colposcopy for diagnostic evaluation, including HPV combined with liquid-based cytology (LBC) cotesting, HPV testing with genotyping and reflex cytology (i.e., primary HPV testing), and cytology alone. Cytology-based CC screening is mostly performed through microscopic observation of cervical cell morphology by cytologists or cytotechnologists with high specificity [5]. However, in low- and middle-income areas, there is a relative shortage of cytotechnologists or cytologists [6]. Furthermore, the HPV test has a slightly higher sensitivity, and some countries are moving towards the HPV test as the primary screening method or HPV and cytology cotesting [7, 8]. However, the implementation of primary HPV screening may result in increased referrals to colposcopy [9].

Given the shortage of cytologists in most developing countries, the popularization of cancer screening is challenging. At present, China has a large rural population that is relatively lacking in medical resources and therefore has had difficulty implementing successful CC screening strategies. Recently, artificial intelligence (AI) technologies based on deep-learning algorithms have been developed in the field of medical diagnostics [10–13]. The convolutional neural network (ConvNet) framework performs exceptionally well for high-dimensional data, as it learns the underlying complex functions within the data empirically and shows better performance than traditional machine learning algorithms [14]. Previous studies have shown that AI-assisted technology might be used for segmentation of the cytoplasm and identification of cervical epithelial dysplasia [15–17]. However, the performance of AI-assisted cytology in clinical screening strategies is still unclear.

In this study, we developed an AI-assisted cytology (AI) system based on a ConvNet and evaluated the system in a CC high-risk population screening program in China. We assessed the effectiveness of AI-assisted cytology as a single or combined screening method.

## Methods

### Study design and inclusion criteria

This study was designed to evaluate AI-assisted cytology for the detection of LSIL, HSIL and worse histology and compare it to LBC with manual reading. We collected a total of 1316 LBC (ThinPrep®) slides from women who underwent cytology, HPV DNA detection and colposcopy at the Department of Obstetrics and Gynecology of the First Medical Center of the Chinese PLA General Hospital (PLAGH) from June 2018 to December 2020. All women were diagnosed by colposcopy-directed biopsy. We obtained patient characteristics and recorded the results of the cytology (both manual and AI-assisted reading) and HPV tests. Diagnoses were made again from all LBC slides using the CC Cell Image Analysis System (CIAS), an AI-assisted cytology system developed by iDeepWise Artificial Intelligence Robot Technology (Beijing) Co. This study was conducted in accordance with the Declaration of Helsinki. The protocol and procedure of this study were approved by the Ethical Committee of the PLAGH. Written consent was obtained from all participants.

### Exclusion criteria

**Fig. 1:**
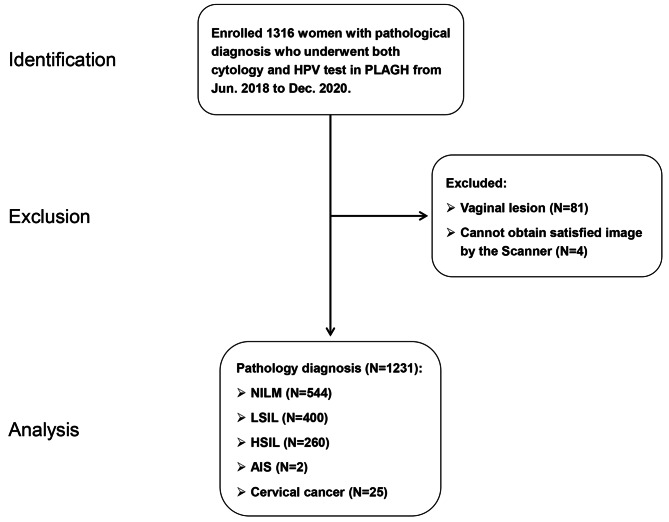
Patient selection flowchart

Patients who had previous cervical lesions and/or had undergone previous cervical treatment (LEEP, conization, photodynamic therapy, etc.) were not eligible, nor were those who had undergone hysterectomy or pelvic radiotherapy, those who were pregnant, those who had vaginal lesions, those with poor-quality LBC slides, or those who were lost to follow-up. Ultimately, we excluded 81 cervical samples from patients with vaginal lesions. Four LBC slides with poor-quality or invalid images following scanning by the AI scanner were excluded (Fig. 1).

### Cytology

LBC slides were prepared using a cytology specimen in sample preservation solution using Papanicolaou staining. The slides were then interpreted by skilled cytologists according to the Bethesda 2001 classification system [18]. Atypical squamous cells of undetermined significance or worse (ASC-US+) were considered abnormal cells [19] and were reviewed by two skilled cytologists. If there was a disagreement in their findings, the diagnosis of the senior cytologist was ultimately selected as the histology result.

### High-risk HPV (HR-HPV) DNA detection

Two systems were used for HPV DNA detection, including the Hybrid Capture 2 (HC2) system (Digene Corporation, Gaithersburg, Md.) for 13 h-HPV genotypes (HPV16, HPV18, HPV31, HPV33, HPV35, HPV39, HPV45, HPV51, HPV52, HPV56, HPV58, HPV59 and HPV68) and the HR-HPV Genotyping Real Time PCR Kit (Shanghai ZJ Bio-Tech Corporation, Shanghai) for 13 h-HPV genotypes and 2 low-risk HPV genotypes (HPV6, HPV11).

### AI-assisted cytology diagnosis

**Fig. 2:**
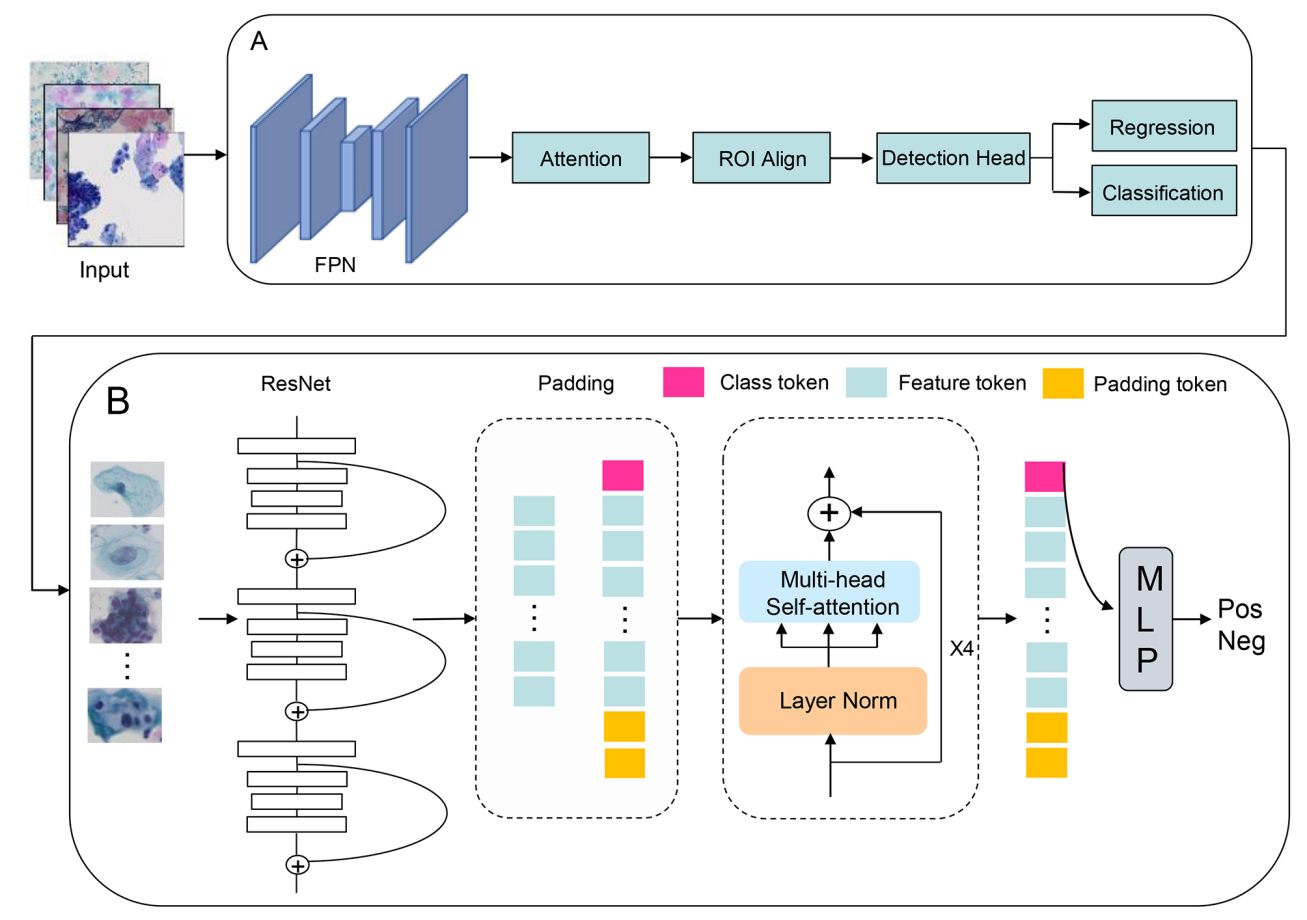
Algorithm framework. **A** Cell detection and cell classification modules. **B** Global interpretation module

The algorithm framework consists of three main modules, including a cell detection module, a cell classification module, and a global interpretation module. The cell detection module locates and identifies potentially abnormal cells in cervical cell images. To contend with similarities in cell morphology in cervical cell images, the attention module, added within the cell detection model, determines the “Attention” between cell features in the embedding space, enhancing the original features and building connections between cells for improving the ability of the model to recognize different cell categories (Fig. 2A). Finally, the global interpretation module is used to conduct an overall analysis of all potentially abnormal cells identified in the LBC slides; the characteristics of different cells are subsequently compared and analysed. In this algorithm, the sequences containing suspected pathological cells identified with the cell detection and cell classification modules are equivalent to video frames. Combined with the overall information from the video interpreting technology modelling image sequence, the sequence features are modelled by a Transformer, and the global diagnosis results of the smears are finally obtained by the classifier and reported as a grade (Fig. 2B). The backbone of the model includes ResNet50 for extracting diagnostic cell features from the smear, which are then integrated through a Transformer layer and multilayer perceptron (MLP) layers to calculate the typicality of lesions in the cell sequence and provide a final qualitative output for the case.

**Fig. 3:**
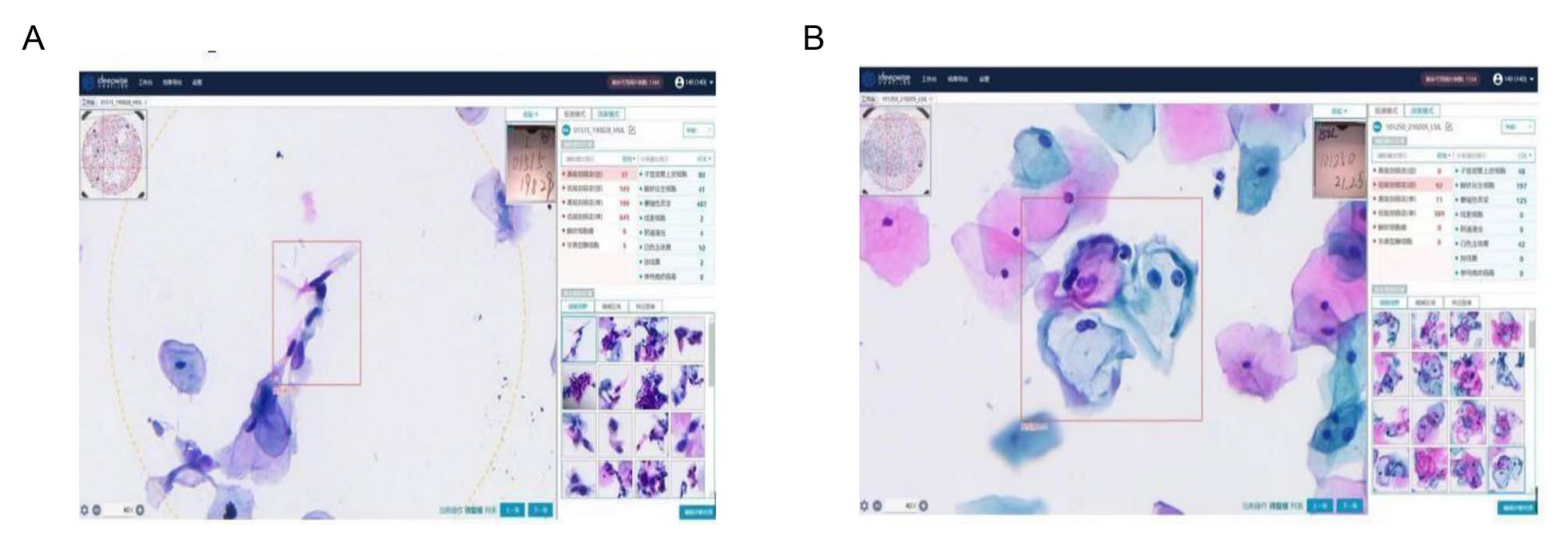
**A** iDeepWise-diagnosed HSIL slide. **B** iDeepWise-diagnosed LSIL slide

The LBC slides for AI analysis were submitted to the iDeepWise Company, where they were placed in the slide holders of the scanner. The Motic EasyScanner scanning software was opened on the computer, and “Scan” was clicked to achieve autofocus and automatic scanning. The software digitizes the slides and generates files in mdsx format on a local computer. Then, the AI analysis software of iDeepWise CIAS was opened, in which clicking on the “Slide Import” function allowed selection of the slide digital image storage folder. Each slide took approximately 80 s to be completely analysed. For the completed slide, the software automatically provides analysis results such as negativity and positivity, The Bethesda System (TBS) grade, a description of the interpretation result, and the slide scanning quality. By clicking “Review”, one can review the details of the current slide to view specific information and suspicious views of the AI analysis. The instrument was operated by professionals from iDeepWise. HSILs (Fig. 3A) and LSILs (Fig. 3B) were diagnosed by the AI system.

### Colposcopy

The surgeries were performed by skilled physicians in the Department of Obstetrics and Gynecology of the First Medical Center of the Chinese PLAGH. During the operation, a speculum was gently placed into the vagina to fully expose the cervix and cervical fornix. The shape, size, colour, presence of erosion, leucoplakia, vegetation, and secretions of the cervix were visually examined. The surfaces of the cervix and vagina were wiped with saline cotton balls to remove mucus and secretions, and the epithelial and subcutaneous vascular structures were observed. Cotton balls soaked in 3–5% acetate were used to swab the surfaces of the cervix and vagina, after 1 min, the surfaces were assessed again for at least 2–3 min. White light was applied to examine the cervix. Changes in the acetowhite epithelium, including mosaicism, blood vessels, gland openings and crypts, were observed. Then, the cervix and vagina were stained with iodine, and any abnormal areas were biopsied, ensuring that at least the epithelium and stroma were sampled. Each biopsy specimen was reviewed by at least two independent pathologists.

### Statistical analysis

The positive interpretation standards are summarized in Table 1. HSIL and LSIL were separately used as the detection thresholds and endpoints for the analysis of sensitivity and specificity. SPSS 22.0 software was used for statistical analysis. Means, medians, and standard deviations are reported for continuous variables. Differences in proportions were assessed using Pearson’s Chi-square test for independent variables. A *P* value < 0.05 was considered to indicate statistical significance. Receiver operating characteristic (ROC) curve analysis, including calculation of the sensitivity and specificity, was conducted.

**Table 1:** Positive interpretation standards

Screening approach	Positive Standards
**AI**	AI-C suggests abnormal cytology
**LBC**	LBC ≥ ASCUS
**HPV**	HR-HPV+; LR-HPV
**LBC + HPV**	HPV16/18+; HR-HPV + and LBC ≥ ASC-US; LBC ≥ ASC-H
**AI + LBC**	LBC ≥ ASC-H; AI + and LBC ≥ ASC-US
**AI + HPV**	HPV 16/18+; AI + and HPV+
**HPV Seq LBC**	HPV16/18+; HR-HPV + and LBC ≥ ASC-US

## Results

### Baseline patient characteristics

**Fig. 4:**
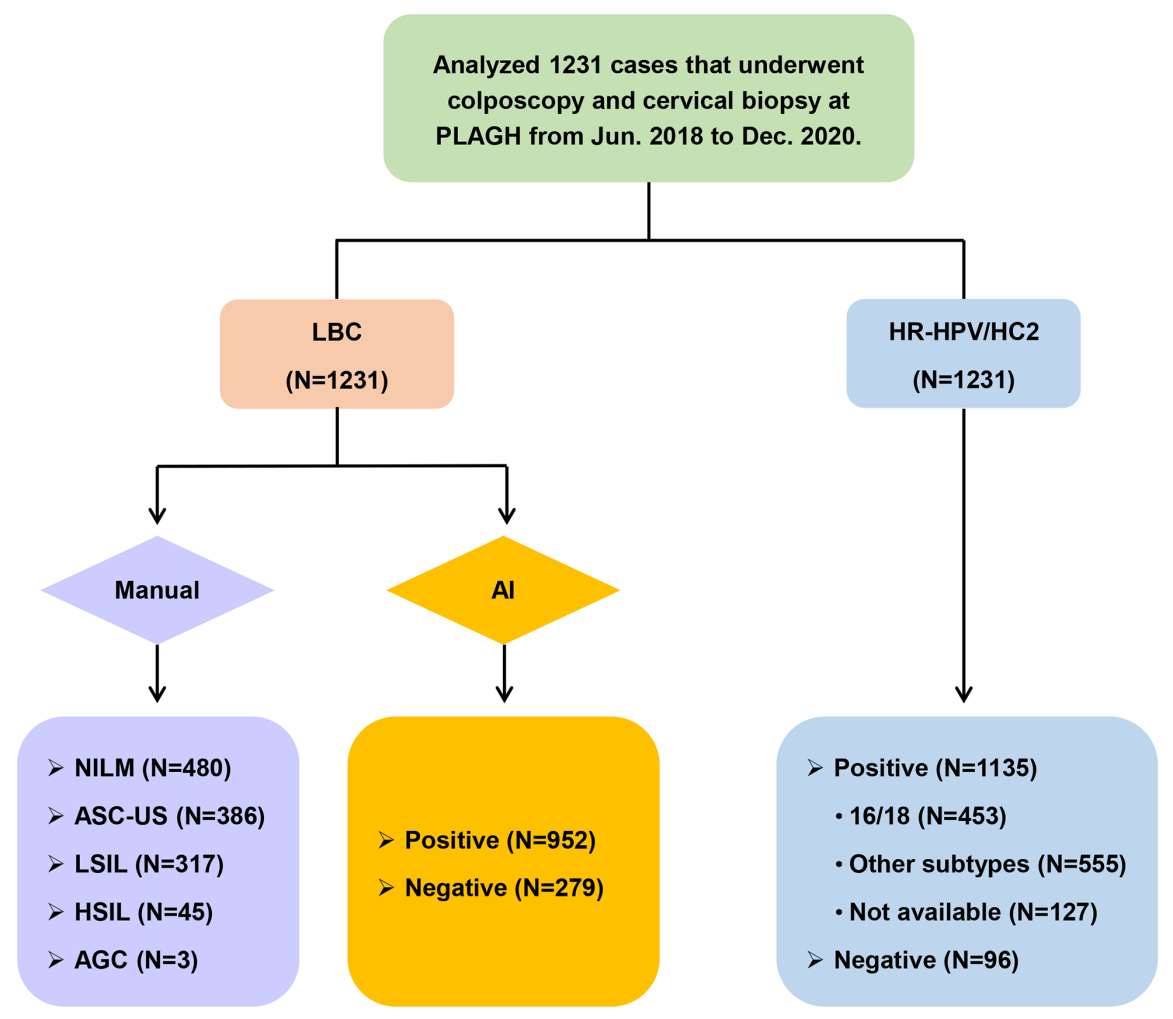
Flow chart of the study design and population

A total of 1316 LBC slides were collected for AI-assisted cytological diagnosis. After excluding 81 slides with vaginal lesions and 4 slides with poor LBC-slide image quality, 1231 slides were included in the analysis (Fig. 4). The demographic and clinical characteristics of the patients are summarized in Table 2. The results of colposcopy and biopsy indicated that 44.19%, 32.50%, 21.12%, 0.16% and 2.03% of the patients in our cohort were negative for intraepithelial lesions or malignancies (NILM) or positive for LSIL, HSIL, AIS and CC, respectively. The positive rates of AI-assisted cytology (AI), LBC testing, HPV testing, LBC and HPV cotesting (LBC + HPV), AI and LBC cotesting (AI + LBC), AI and HPV cotesting (AI + HPV), and HPV followed by LBC testing (HPV Seq LBC) were 77.34%, 61.01%, 92.20%, 79.20%, 59.06%, 81.80%, and 76.69%, respectively. There were significant differences in age and the positive rates from AI, LBC testing, HPV testing, LBC + HPV, AI + LBC, AI + HPV, and HPV Seq LBC among the five patient groups (*P* < 0.05).

**Table 2:** Demographics and clinical characteristics of women

Screening	Histological diagnosis					
	NILM	LSIL	HSIL	AIS	CC	*P* value
No. of patients (*N* = 1231)	544 (44.19%)	400 (32.50%)	260 (21.12%)	2 (0.16%)	25 (2.03%)	
**Age (years old)**						< 0.001
< 25 (*N* = 29)	16 (55.17%)	8 (27.59%)	5 (17.24%)	0 (0.00%)	0 (0.00%)	
25–30 (*N* = 165)	65 (39.39%)	54 (32.73%)	42 (25.45%)	1 (0.61%)	3 (1.82%)	
31–35 (*N* = 212)	69 (32.55%)	81 (38.21%)	60 (28.30%)	1 (0.47%)	1 (0.47%)	
36–40 (*N* = 200)	89 (44.50%)	64 (32.00%)	44 (22.00%)	0 (0.00%)	3 (1.50%)	
41–45 (*N* = 173)	83 (47.98%)	50 (28.90%)	37 (21.39%)	0 (0.00%)	3 (1.74%)	
46–50 (*N* = 151)	70 (46.36%)	53 (35.10%)	24 (15.89%)	0 (0.00%)	4 (2.65%)	
51–55 (*N* = 114)	57 (50.00%)	37 (32.45%)	19 (16.67%)	0 (0.00%)	1 (0.88%)	
> 55 (*N* = 187)	95 (50.80%)	53 (28.34%)	29 (15.51%)	0 (0.00%)	10 (5.35%)	
**AI**						< 0.001
Positive (*N* = 952, 77.34%)	341 (35.82%)	336 (35.29%)	250 (26.26%)	2 (0.21%)	23 (2.42%)	
Negative(*N* = 279, 22.66%)	203 (72.76%)	64 (22.94%)	10 (3.58%)	0 (0.00%)	2 (0.72%)	
**LBC**						< 0.001
NILM (*N* = 480, 38.99%)	276 (57.50%)	118 (24.58%)	76 (15.83%)	2 (0.42%)	8 (1.67%)	
ASC-US (*N* = 386, 31.36%)	166 (43.00%)	135 (34.97%)	79 (20.47%)	0 (0.00%)	6 (1.56%)	
LSIL (*N* = 317, 25.75%)	95 (29.97%)	141 (44.48%)	78 (24.60%)	0 (0.00%)	3 (0.95%)	
HSIL (*N* = 45, 3.66%)	6 (13.33%)	5 (11.11%)	26 (57.78%)	0 (0.00%)	8 (17.78%)	
AGC (*N* = 3, 0.24%)	1 (33.33%)	1 (33.33%)	1 (33.33%)	0 (0.00%)	0 (0.00%)	
**HPV**						0.020
Positive(*N* = 1135, 92.20%)	487 (42.91%)	374 (32.95%)	249 (21.94%)	2 (0.17%)	23 (2.03%)	
16/18 (*N* = 453)	186 (41.06%)	110 (24.28%)	141 (31.13%)	2 (0.44%)	14 (3.09%)	
Others (*N* = 555)	246 (44.32%)	224 (40.36%)	82 (14.78%)	0 (0.00%)	3 (0.54%)	
NA (*N* = 127)	55 (43.31%)	40 (31.50%)	26 (20.47%)	0 (0.00%)	6 (4.72%)	
Negative (*N* = 96, 7.80%)	57 (59.38%)	26 (27.08%)	11 (11.46%)	0 (0.00%)	2 (2.08%)	
**LBC + HPV**						< 0.001
Positive (*N* = 975, 79.20%)	384 (39.38%)	333 (34.15%)	233 (23.90%)	2 (0.21%)	23 (2.36%)	
Negative(*N* = 256, 20.80%)	160 (62.50%)	67 (26.17%)	27 (10.55%)	0 (0.00%)	2 (0.78%)	
**AI + LBC**						< 0.001
Positive (*N* = 727, 59.06%)	254 (34.94%)	276(37.96%)	181(24.90%)	0 (0.00%)	16 (2.20%)	
Negative (*N* = 504, 40.94%)	290 (57.54%)	124(24.60%)	79 (15.67%)	2 (0.40%)	9 (1.79%)	
**AI + HPV**						< 0.001
Positive (*N* = 1007, 81.80%)	401 (39.82%)	337 (33.47%)	244 (24.23%)	2 (0.20%)	23 (2.28%)	
Negative (*N* = 224, 18.20%)	143 (63.84%)	63 (28.13%)	16 (7.14%)	0 (0.00%)	2 (0.89%)	
**HPV Seq LBC**						< 0.001
Positive (*N* = 944, 76.69%)	371 (39.30%)	321 (34.00%)	229 (24.26%)	2 (0.21%)	21 (2.23%)	
Negative (*N* = 287, 23.31%)	173 (62.28%)	79 (27.53%)	31 (10.80%)	0 (0.00%)	4 (1.39%)	

### Performance of different screening strategies at the threshold of HSIL

Currently, sensitivity (Se) and specificity (Sp) are the most widely used metrics for assessing the performance of diagnostic tests [20]. Thus, we calculated the Se and Sp of the AI, LBC, HPV, LBC + HPV, AI + LBC, AI + HPV and HPV Seq LBC screening strategies at the HSIL + threshold and obtained the following results: AI (Se, 95.80%; Sp, 28.30%), LBC (Se, 70.00%; Sp, 41.70%), HPV (Se, 95.00%; Sp, 8.80%), LBC + HPV (Se, 89.90%; Sp, 24.00%), AI + LBC (Se, 68.30%; Sp, 44.30%), AI + HPV (Se, 93.70%; Sp, 21.80%) and HPV Seq LBC (Se, 87.80%; Sp, 26.70%). These data showed that the Se and Sp of AI alone were superior to those of LBC + HPV cotesting. Compared with the HPV Seq LBC strategy, the AI strategy had a better Se and similar Sp (Table 3).

**Table 3:** Performance of Different Screening Strategies at the threshold of HSIL + in overall patients (*N* = 1231)

	AI	LBC	HPV	LBC + HPV	AI + LBC	AI + HPV	HPV Seq LBC
**Se (%)**	95.80	70.00	95.50	89.90	68.30	93.70	87.80
**Sp (%)**	28.30	41.70	8.80	24.00	44.30	21.80	26.70
**PPV (%)**	28.90	26.80	24.10	26.50	27.10	26.70	26.70
**NPV (%)**	95.70	82.10	86.50	88.70	82.10	92.00	87.80
**FPR (%)**	71.70	58.30	91.20	76.00	55.70	87.20	73.30
**FNR (%)**	4.20	30.00	4.50	10.10	31.70	6.30	12.20
**OA (%)**	44.00	48.30	29.00	39.40	50.00	38.60	40.90
**PLR**	1.34	1.20	1.05	1.18	1.23	1.20	1.20
**NLR**	0.15	0.72	0.52	0.42	0.72	0.29	0.46
**YI**	0.241	0.117	0.043	0.139	0.126	0.155	0.145

AI, Artificial Intelligence-assisted cytology; FNR, False Negative Rate; FPR, False Positive Rate; HPV, Human papillomavirus; HSIL+, High grade Squamous Intraepithelial Lesion or higher; LBC, liquid-based cytology; NLR, Negative Likelihood Ratio; NPV, Negative Predictive Value; OA, Overall Accuracy; PLR, Positive Likelihood Ratio; PPV, Positive Predictive Value; Se, Sensitivity; Sp, Specificity; YI, Youden Index

Other indicators derived from Se and Sp that are important for evaluating the authenticity of diagnostic tests are the positive predictive value (PPV), negative predictive value (NPV), false-negative rate (FNR), false-positive rate (FPR), overall accuracy (OA), positive likelihood ratio (PLR), negative likelihood ratio (NLR) and Youden index (YI). We also calculated these indicators for the AI, LBC, HPV, LBC + HPV, AI + LBC, AI + HPV and HPV Seq LBC screening strategies at the HSIL + threshold (Table 3). The PPV of the AI strategy (28.90%) was the highest among the investigated strategies. Furthermore, among all screening strategies, the YI of the AI strategy (0.241) was also the highest. Overall, AI-alone screening was the most authoritative method for diagnosing HSILs, improving the accuracy of colposcopy diagnosis.

### Performance of different screening strategies at the LSIL threshold

To assess the performance of these different screening strategies at the LSIL threshold, we calculated the Se, Sp, PPV, NPV, FPR, FNR, OA, PLR, NLR and YI. The Se and Sp of the AI, LBC, HPV, LBC + HPV, AI + LBC, AI + HPV and HPV Seq LBC screening strategies at the LSIL + threshold are as follows: AI (Se, 88.90%; Sp, 37.30%), LBC (Se, 70.30%; Sp, 50.70%), HPV (Se, 94.30%; Sp, 10.50%), LBC + HPV (Se, 86.00%; Sp, 29.40%), AI + LBC (Se, 68.10%; Sp, 53.30%), AI + HPV (Se, 88.20%; Sp, 26.30%) and HPV Seq LBC (Se, 83.40%; Sp, 31.80%). These data show that the Se and Sp of AI alone were superior to those of LBC + HPV cotesting. Compared with the HPV Seq LBC strategy, the AI strategy also had better Se and Sp (Table 4). In addition, we also calculated other parameters at the LSIL + threshold and found that the PPV of the AI + LBC strategy (91.90%) was the highest among all the strategies. Additionally, among all screening strategies, the YI of the AI (0.262) at the LSIL + threshold was the highest (Table 4). Overall, AI-alone screening was the most authoritative method for diagnosing LSILs.

**Table 4:** Performance of Different Screening Strategies at the threshold of LSIL + in overall patients (*N* = 1231)

	AI	LBC	HPV	LBC + HPV	AI + LBC	AI + HPV	HPV Seq LBC
**Sensitivity (%)**	88.90	70.30	94.30	86.00	68.10	88.20	83.40
**Specificity (%)**	37.30	50.70	10.50	29.40	53.30	26.30	31.80
**PPV (%)**	64.20	64.30	57.10	60.60	91.90	60.20	60.70
**NPV (%)**	72.80	57.50	59.40	62.50	40.20	63.80	60.30
**FPR (%)**	62.70	49.30	89.50	70.60	46.70	73.70	68.20
**FNR (%)**	11.10	29.70	5.70	14.00	31.90	11.80	16.60
**OA (%)**	66.10	61.70	57.30	61.00	61.60	60.80	60.60
**PLR**	1.42	1.43	1.05	1.22	1.46	1.20	1.22
**NLR**	0.30	0.59	0.54	0.48	0.60	0.45	0.52
**YI**	0.262	0.21	0.048	0.154	0.214	0.145	0.152

AI, artificial intelligence-assisted cytology; FNR, False Negative Rate; FPR, False Positive Rate; HPV, Human papillomavirus; LBC, liquid-based cytology; LSIL+, Low grade Squamous Intraepithelial Lesion or higher; NLR, Negative Likelihood Ratio; NPV, Negative Predictive Value; OA, Overall Accuracy; PLR, Positive Likelihood Ratio; PPV, Positive Predictive Value; Se, Sensitivity; Sp, Specificity; YI, Youden Index

### ROC curve analysis for the different screening strategies

**Fig. 5:**
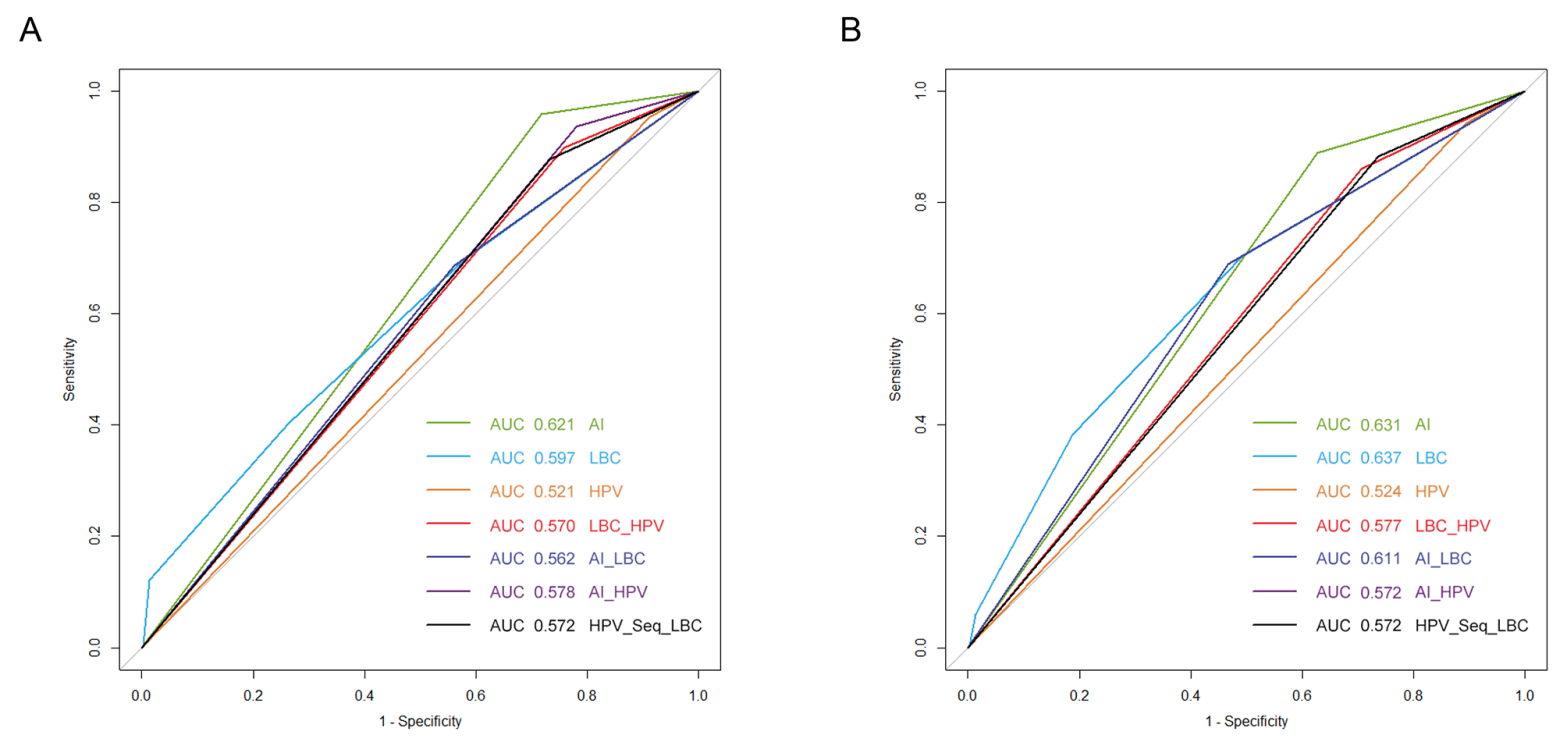
ROC curves of the different screening strategies after setting the endpoints to HSIL (**A**) and LSIL (**B**). The lines represent the ROC curves for the AI-C, LBC, HPV, LBC + HPV, AI + LBC and AI + HPV strategies

The ROC curve can be interpreted as a diagnostic tool for comparing true sensitivity and 1- specificity, in which only curves lying above the diagonal (identity) line represent good results. ROC curve analysis revealed that the AUCs of the AI, LBC, HPV, LBC + HPV, AI + LBC, AI + HPV and HPV Seq LBC strategies at the HSIL threshold were 0.621, 0.597, 0.521, 0.570, 0.562, 0.578 and 0.572, respectively (Table 5; Fig. 5A). The AI strategy achieved the best result, with an AUC value of 0.621, whereas HPV testing achieved the worst result, with an AUC value of 0.521. The AUC of the AI strategy was significantly different from that of all other strategies (except the LBC strategy) at the HSIL threshold. Similarly, the AUCs of the AI, LBC, HPV, LBC + HPV, AI + LBC, AI + HPV and HPV Seq LBC strategies at the LSIL threshold were 0.631, 0.637, 0.524, 0.577, 0.611, 0.572 and 0.576, respectively (Table 5; Fig. 5B). Here, the LBC strategy achieved the best result, with an AUC value of 0.637, whereas HPV testing achieved the worst result, with an AUC value of 0.524. Moreover, the AUCs of the AI and LBC strategies were similar (0.631 and 0.637, respectively). The AUC of the AI strategy was significantly different from that of the other strategies (except the LBC and AI + LBC strategies) at the LSIL threshold. These results further demonstrate the superiority of AI-alone screening over other screening strategies in diagnosing HSILs and LSILs.

**Table 5:** Statistical analysis of AUC.

Variable	AUC	95% CI	*P* value
**HSIL threshold**			
AI	0.621	0.602–0.639	-
LBC	0.597	0.560–0.635	0.2172
HPV	0.521	0.506–0.536	< 0.0001
LBC + HPV	0.570	0.548–0.592	< 0.0001
AI + LBC	0.562	0.531–0.594	< 0.0001
AI + HPV	0.578	0.559–0.597	< 0.0001
HPV Seq LBC	0.572	0.549–0.596	0.0001
**LSIL threshold**			
AI	0.631	0.608–0.655	-
LBC	0.637	0.608–0.666	0.6897
HPV	0.524	0.509–0.540	< 0.0001
LBC + HPV	0.577	0.554-0.600	< 0.0001
AI + LBC	0.611	0.584–0.638	0.0678
AI + HPV	0.572	0.550–0.595	< 0.0001
HPV Seq LBC	0.576	0.552-0.600	< 0.0001

## Discussion

In this study, we compared single screening strategies (AI, LBC and HPV) and combined screening strategies (LBC + HPV, AI + LBC, AI + HPV and HPV Seq LBC) for diagnosing LSILs, HSIL, and CC. We revealed that the Se, Sp and YI of AI alone were superior to those of other screening strategies in diagnosing HSILs and LSILs. Furthermore, ROC curve analysis demonstrated that AI was the most efficient screening strategy for diagnosing HSILs and LSILs.

HPV and LBC tests have been applied in clinical practice for screening CC [21]. However, these examinations have numerous shortcomings, including a dependence on the subjective experience of the operator, substantial inter- and intraoperator variability, a shortage of experienced colposcopists, the need to undergo colposcopy training courses, uniform diagnostic standards and strict quality control [13]. Recently, HPV testing has been recommended as a primary screening method for CC due to its ease of operation and high sensitivity [22]. However, the specificity of primary HPV testing is relatively low, and so this strategy may lead to many unnecessary colposcopy referrals. Fortunately, AI technology has been widely used in medical diagnosis.

However, few studies have incorporated AI-assisted cytology into clinical screening strategies for CC. To date, the development of AI-assisted medical diagnostics and AI-assisted cytology has been reported to facilitate screening for CC. Some researchers have confirmed that AI-assisted cytology could improve the sensitivities in detecting LSIL and HSIL and can achieve similar sensitivity and specificity to those of cytologists in the referral population [23]. A previous study in which researchers evaluated the performance of an AI-assisted system in detecting cervical intraepithelial lesions (CINs) or CCs revealed that AI-assisted reading had greater specificity and similar sensitivity to manual reading [24]. In another study, cytology slides considered negative during manual reading were excluded by an AI-assisted cytology system, and the efficiency of CIN2 + detection was improved [12]. Another study showed that the automated visual evaluation of cervical images was more accurate than cervigram results [25]. The AUC of a deep-learning model in detecting cervical lesions was 0.947, with 88.2% specificity and 85.2% sensitivity [26]. Likewise, researchers have developed a novel deep-learning image analysis platform that can count p16/Ki-67 dual-stained cells and dramatically improved the efficiency of CC screening over current methods [27]. Nevertheless, whether AI can replace the predominance of LBC testing in the field of cervical cytology screening, whether the HPV test can omitted, and whether it is feasible to perform AI screening alone or in combination with LBC testing remain to be further explored. Cytology-based cervical screening has been a public health strategy in the guidelines of many different countries, including China [28].

In this study, the CIAS cervical cytology screening system developed by the iDeepWise Company, which can locate and identify the cells of suspected lesion in cervical slides and provide negative or positive for review and interpretation by doctors, was used to assess the capabilities of AI-assisted cytology. The CIAS cervical cytology screening system, which provides medical interpretability, was constructed by combining doctors’ diagnostic criteria, cytology domain knowledge and artificial intelligence knowledge. The system is mainly composed of an image quality evaluation module, a cell detection module, a cell classification module, and a global interpretation module. It can effectively identify complex scenarios such as the presence of pathogenic microorganisms, glandular abnormalities, and infectious and reactive changes. Specifically, the image quality evaluation module can be used to evaluate the scanning and production quality of the slide to avoid false negatives caused by the generation of unsatisfactory samples. Additionally, the cell detection module reduces positioning errors caused by unclear cell boundaries and large size differences through multiscale model training and the use of attention mechanisms, leading to greater recognition accuracy for different pathological cell types with similar cell morphologies. The cell classification module is divided into a cluster classification model and a single classification model. In the cluster classification model, a graph convolution neural network model was designed to model the relationships between cells to improve the recognition accuracy for clustered cells. For the single-cell classification model, an attention mechanism is used to effectively leverage the feature information of the nucleus by explicitly extracting nuclear features, highlighting the importance of nuclei in the classification of individual cells. The global interpretation module extracts the implicit correspondence between the detected pathological cells and produces negative or positive results as well as grading results for the entire slide.

The application of AI in cervical cytology holds immense promise for improving the accuracy, efficiency, and accessibility of CC screening programs. By automating the screening process, enhancing accuracy and consistency, integrating with imaging technologies, and predicting disease progression, AI can revolutionize the field and contribute to the early detection and prevention of CC. In our study, the AI screening scheme was more beneficial for patients than the traditional combination screening scheme, LBC + HPV cotesting screening. However, it is crucial to address the challenges and ethical considerations associated with AI implementation to ensure its responsible and ethical use. With further research and development, AI has the potential to transform cervical cytology and significantly reduce the burden of CC worldwide.

This study had several limitations. First, few healthy women were included in the study, mainly because the gold standard of this study was the histopathological diagnosis, which is difficult to obtain in the general population. The sensitivity and specificity were estimated in a referral population but do not necessarily apply to the general population, for which the specificity is greater. Second, our evaluation is limited by the number of glandular lesions, and it is difficult to demonstrate the advantages of AI in adenocarcinoma screening. However, we found that only two cases of AIS were misdiagnosed as NILM according to manual LBC, while AI indicated abnormal cytological results in both cases. Third, we included participants and collected samples from one hospital, and slide preparation and scanning were performed in one laboratory. The database of the iDeepWise Company consists of slides from all over the country, covering different pieces of scanning equipment, different production methods, different consumables, and different patient ages, ensuring a highly diverse dataset distribution. Data annotation was performed by experienced professional doctors through three-level quality control annotation to ensure that the dataset was of high annotation quality. In the future, we will conduct multicentre studies by including different slide processing laboratories and scanners.

The strengths of this study were that AI-assisted cytology systems had better sensitivity and specificity for detecting HSILs or higher-grade lesions. In addition, the clinical application of AI-assisted cytology systems will effectively alleviate the shortage of cytology pathologists in China and improve the coverage and efficiency of cervical cancer screening. This machine learning system can also be improved by conducting large-scale prospective data validation in the future to increase the system’s screening sensitivity and specificity, reduce the risk of missed diagnoses due to human factors, and contribute to achieving the WHO’s goal of eliminating cervical cancer by 2030.

In summary, this study compared AI-assisted cytology with other screening strategies, including LBC-alone screening, HPV-alone screening, and LBC + HPV, AI + LBC, AI + HPV and HPV Seq LBC screening, demonstrating that AI-assisted cytology is comparable to other screening methods while offering superior diagnostic efficacy. Therefore, AI-assisted cytology is a novel primary CC screening model that reduces the size of the high-risk referral population and improves the accuracy of colposcopy diagnosis and cervical biopsy.

## Conclusions

In this retrospective study, we demonstrated that the AI-assisted cytology screening system had excellent diagnostic efficacy both alone and in combination with other, existing CC screening strategies. AI-assisted cytology screening has the potential to be widely used as a primary strategy for CC.

## Data Availability

The datasets used and/or analysed during the current study are available from the corresponding author on reasonable request.

## Abbreviations

AGC: Atypical glandular cells
AI: Artificial intelligence-assisted cytology
AIS: Adenocarcinoma in situ
ASC-H: Atypical squamous cells – cannot exclude high grade squamous intraepithelial lesions
CC: Cervical cancer
CIAS: Cell image analysis system
FNR: False negative rate
FPR: False positive rate
HPV: Human papillomavirus
HSIL: High-grade squamous intraepithelial lesion
LBC: Liquid-based cytology
LSIL: Low-grade squamous intraepithelial lesion
NILM: Negative for intraepithelial lesions or malignancies
NLR: Negative likelihood ratio
NPV: Negative predictive value
OA: Overall accuracy
PLR: Positive likelihood ratio
PPV: Positive predictive value
ROC: Receiver operating characteristic
Se: Sensitivity
Sp: Specificity
YI: Youden index
